# Potential Hypotheses Predicting Leaf Litter Nitrogen and Phosphorus Patterns at the Global Scale

**DOI:** 10.3390/plants14213356

**Published:** 2025-11-01

**Authors:** Yajun Xie, Jiacheng Yan, Yonghong Xie

**Affiliations:** 1 College of Civil and Environmental Engineering, Hunan University of Technology, Zhuzhou 412007, China; 2 Dongting Lake Station for Wetland Ecosystem Research, Institute of Subtropical Agriculture, The Chinese Academy of Sciences, Changsha 410125, China

**Keywords:** leaf litter N, leaf litter P, soil nutrient, *Temperature* *–Plant Physiological hypothesis*, *Soil Substrate Age hypothesis*, *Species Composition hypothesis*, plant functional type

## Abstract

Climate has shaped green leaf nitrogen (N) and phosphorus (P) patterns through its direct physiological effects (*Temperature–Plant Physiology hypothesis*), indirect pathways involving soil nutrients (*Soil Substrate Age hypothesis*), or vegetation composition (*Species Composition hypothesis*). However, the efficiencies of these hypotheses and the relative importance of the factors involved in predicting leaf litter N and P remain unresolved. We evaluated these hypotheses by analyzing 4657 global observations of leaf litter N and P concentrations and N/P ratios, demonstrating that litter stoichiometries diverged in plant functional types, and that litter N and N/P ratios declined with latitude, while P increased. The validity of each hypothesis in predicting latitudinal patterns of leaf litter P was confirmed, with the *Species Composition hypothesis* being the most effective model; however, all hypotheses failed to predict the litter N. Environmental and biological factors collectively explained over 40% of the variations in litter stoichiometries, with plant functional type, soil pH, and climatic factors being the most important drivers of litter N, P, and N/P ratio, respectively. The fundamentally different control mechanisms of litter stoichiometry patterns compared with those of green leaves challenge the idea that common hypotheses can predict biogeographic patterns across all leaf stages; thus, current litter element biogeochemical models and plant nutrition paradigms require revision.

## 1. Introduction

Litter decomposition represents a critical but poorly understood process, which has consequences for soil organic matter formation, nutrient availability, plant growth, and ecosystem sensitivity to global change [1,2]. The importance of litter quality, particularly its nitrogen (N) and phosphorus (P) concentrations, has been explored extensively as a major control of litter decomposition [3]. Moreover, N and P remain in litter after senescence and eventually return to soil during decomposition, representing a major source of mineral nutrients and heavy metals in most ecosystems [1]. Investigating the relationship between litter N and P and environmental climatic variables will improve our capacity to predict global biogeochemical cycling responses to climate change scenarios [4,5,6].

**Figure 1:**
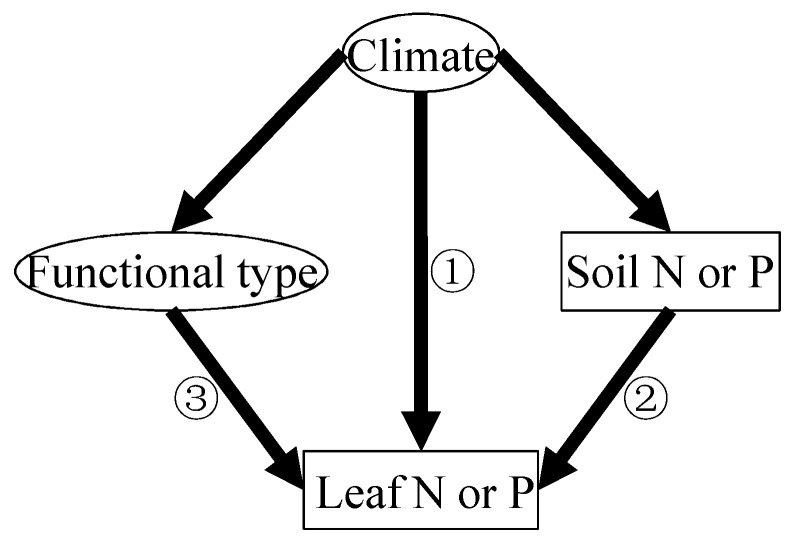
Conceptual framework illustrating three distinct mechanisms governing latitudinal variations in foliar nitrogen (N) and phosphorus (P) concentrations: ① *Temperature-Plant Physiology hypothesis*, ② *Soil Substrate Age hypothesis*, and ③ *Species Composition hypothesis* [7].

The biogeography patterns of leaf N and P are driven collectively in a complex manner by climate-related gradients of plant physiological stoichiometry, soil chemistry (i.e., element concentration and pH), and plant functional types [7,8]. There are three well-established hypotheses explaining the latitudinal pattern in plant N and P that are currently understood in this field [7,8,9]. First, the *Temperature–Plant Physiology hypothesis* proposes a direct climatic effect (Figure 1) [7,8]; under colder conditions, plants elevate foliar N and P concentrations to compensate for reduced metabolic efficiency, as temperature-dependent enzymatic processes involving N-rich proteins and P-rich ribosomal RNA become less efficient [9]. Second, the *Soil Substrate Age hypothesis* emphasizes indirect edaphic controls (Figure 1) [5,8]; being generally more weathered and nutrient-depleted, tropical soils limit plant nutrient uptake compared to younger, less-leached soils at higher latitudes [5]. Third, the *Species Composition hypothesis* highlights biological mediation (Figure 1) [10]; latitudinal shifts in vegetation structure, particularly the predominance of nutrient-poor evergreen species in the tropics versus nutrient-rich deciduous species in temperate zones, amplify these nutrient gradients [10].

These proposed hypotheses originate from controlled experimental studies; therefore, their applicability to natural systems requires rigorous empirical validation [11]. A critical knowledge gap persists in determining the relative predictive power of each hypothesis, as their proposed mechanisms frequently interact and overlap [7]. To date, these hypotheses have been tested primarily on green leaves, with their validity confirmed across scales ranging from regional to global in previous research [12,13]. Specifically, the validity of each hypothesis on a global scale was confirmed, with the exception of the *Soil Substrate Age hypothesis*, which failed to predict leaf N, as the climatic influence on leaf N occurs through a mechanism in opposition to what the hypothesis suggests [13]. However, similar tests have not been conducted on leaf litter nutrients. Due to resorption during the processes of litter formation, litter traits are not a mere legacy of those of green leaves [14], and in turn, the patterns as well as potential hypotheses in leaf litter stoichiometry might be different from those of green leaf stoichiometry [15]. However, currently, no assessment has systematically compared these hypotheses, largely because existing datasets lack the necessary complementary measurements—most notably, comprehensive soil characteristic data [4,5]. Therefore, it is necessary to test these potential hypotheses for leaf litter stoichiometry.

Moreover, leaf litter N and P levels emerge from the complex interactions between biological, climatic, and pedological drivers, including soil chemistry and physical properties [4,16]. Until now, it has been difficult to comprehensively elucidate the factors that govern leaf litter stoichiometry patterns [17,18]. However, the relative contribution of these controlling factors in shaping worldwide foliar N and P patterns remains insufficiently characterized [5].

In this study, we present a novel global-scale investigation integrating leaf litter stoichiometry (namely concentrations of both N and P and mass ratios of N/P), plant functional type (including non-seed plants, grasses, herbs, broadleaf woody, and conifers), soil properties (namely soil N, soil P, and soil pH), and climatic variables (namely latitude, mean annual temperature (MAT), and mean annual precipitation (MAP)) to elucidate the drivers of leaf litter elemental composition. We aim to answer the following questions: (1) How does leaf litter stoichiometry vary across different plant functional types? (2) Which hypotheses have the best fit in predicting global patterns of leaf litter N and P? (3) Among each factor, what are the strongest determinants explaining leaf litter stoichiometry?

## 2. Results

### 2.1. Leaf Litter Stoichiometry at Global Scale

The mean N and P concentrations of leaf litter were 1.200% (*n* = 4594, *SD* = 0.738%) and 0.088% (*n* = 3360, *SD* = 0.079%) (Figure S1), respectively, while the stoichiometric N/P ratio across all species averaged 21.47 (*n* = 3426, *SD* = 18.84), indicating a predominant P limitation globally [19]. Leaf litter stoichiometric variability differed substantially, with N/P showing the greatest relative variation (*CV* = 180.89%), followed by P (*CV* = 90.11%) and N (*CV* = 61.56%).

**Figure 2:**
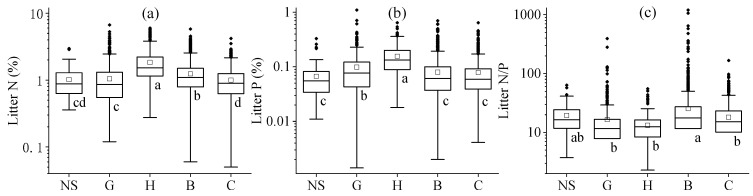
Global variation in leaf litter N (**a**) and P (**b**) concentrations and mass ratios of N/P (**c**) across major plant functional groups. Leaf litter stoichiometries were compared among five vegetation types: non-seed plants (NS), grasses (G), herbs (H), broadleaf woody (B), and conifers (C). Statistical differences (LSD post hoc test, *α* = 0.05) are indicated by superscript letters, with different letters denoting significant differences.

The leaf litter stoichiometries showed substantial differences among various plant functional types (Figure 2 and Table S1, *p* < 0.05 or *p* < 0.01). In general, herb litter was richest in N and P, but lowest in terms of its N/P ratio. In contrast, conifers and non-seed plants had the lowest N and P contents, respectively (Figure 2, *p* < 0.05). Broadleaf trees showed the highest N:P ratios; among the woody plants, conifers generally had nutrient-poor leaf litter in contrast with the broadleaf plants (Table S1, *p* < 0.05 or *p* < 0.01).

### 2.2. Variation in Leaf Litter Stoichiometry with Latitude and Climate

**Figure 3:**
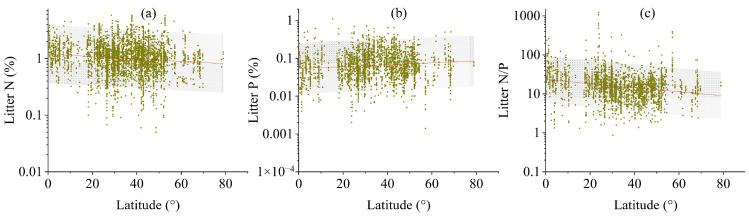
Latitudinal gradients in leaf litter N (**a**) and P (**b**) concentrations and mass ratios of N/P (**c**) across global ecosystems. All values were presented on log_10_-transformed scales. Each data point represents site-level measurements of leaf litter chemistry plotted against absolute latitude.

Across the global scale, distinct latitudinal trends emerged in leaf litter stoichiometry (Figure 3, *p* < 0.01 or *p* < 0.05). The concentration of leaf litter P escalated progressively from the equatorial regions towards the polar zones (Figure 3b, *p* < 0.01); in contrast, the concentrations of N and the N/P ratios in leaf litter exhibited an inverse latitudinal trend (Figure 3a,c, *p* < 0.01). These latitudinal patterns generally held consistent even when the analysis was conducted separately for each of the five major plant functional types (Figure S2, *p* < 0.01 or *p* < 0.05).

### 2.3. Relationship Between Leaf Litter Stoichiometry and Potential Driving Factors

**Figure 4:**
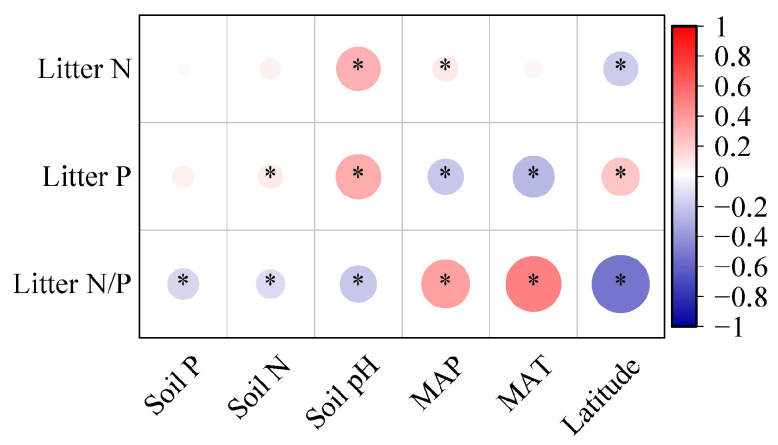
Correlation analysis between leaf litter stoichiometries and environmental factors. The heatmap visualization quantifies relationships between leaf litter stoichiometries (namely concentrations of N and P and N:P ratios), climatic variables (namely latitude, mean annual temperature (MAT), and mean annual precipitation (MAP)), and pedological characteristics (namely soil N, soil P, and soil pH). Asterisks indicate statistically significant correlations (*α* = 0.05 threshold).

When analyzing all species, it was found that the leaf litter P concentration had a positive correlation with soil N levels, while the leaf litter N/P ratio was negatively correlated with soil nutrients (Figure 4, *p* < 0.05). Leaf litter N and P concentrations were positively correlated with the soil pH, while the N/P ratio was negatively correlated with soil pH (Figure 4, *p* < 0.05). Regarding the relationship between climatic factors (MAT and MAP) and various leaf litter stoichiometries, different trends emerged; specifically, the leaf litter N concentrations and N/P ratios were negatively associated with latitude and positively with MAP, while P concentrations in leaves showed contrary correlations with these climatic factors (Figure 4, *p* < 0.05).

### 2.4. Determinants of Leaf Litter N and P at Global Scale

**Figure 5:**
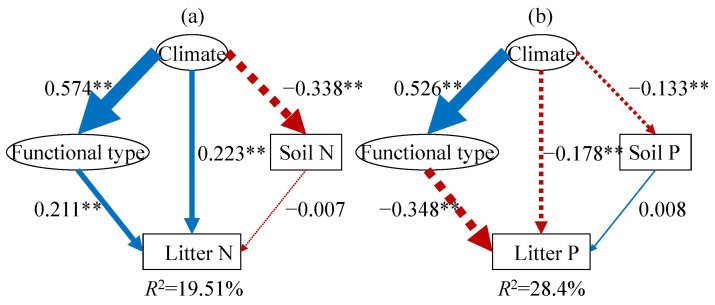
SEM illustrating the existing hypotheses concerning the biogeographical patterns of leaf litter N (**a**) and P (**b**). Positive effects are indicated by blue solid arrows, while red dashed arrows denote significant negative effects. The thickness of these arrows reflects the strength of the relationships. The numbers on the arrows represent standardized path coefficients, which quantify the magnitude of the relationships. The climate variable is a composite measure incorporating mean annual temperature and precipitation, and the functional type variable aggregates data from non-seed plants, grasses, herbs, broadleaf woody, and conifers. The *R*^2^ values illustrate the proportion of variance accounted for by the models. ** represents *p* < 0.01.

We further employed structural equation models (SEMs) to clarify the relative importance and the direct and indirect effects of climatic factors, plant functional types, and soil nutrients on leaf litter N and P (Figure 5). The SEM-derived models explained 19.51% and 28.40% of the variance in leaf litter N and P concentrations, respectively (Figure 5). The climate-related composite variable pathway (comprising MAT and MAP) revealed its overall impact on leaf litter elements. In accordance with the potential hypothesis (Figure 1), plant functional type and climate predicted the leaf litter P fairly well (Figure 5b); specifically, both plant functional type and climate had a negative impact on leaf litter P (Figure 5b, *p* < 0.01). Among these hypotheses, the *Species Composition hypothesis* provided the best predictive model for leaf litter P (Figure 5b); however, in contrast to the potential hypothesis, plant functional type and climate were positively related to the leaf litter N concentration, while soil N had no significant impact on leaf litter N (Figure 5a). Therefore, the potential hypothesis failed to predict the leaf litter N.

**Figure 6:**
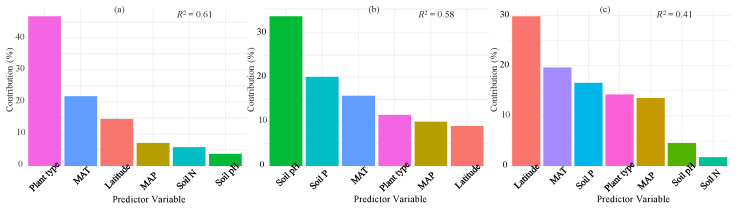
The percentage of variance in leaf litter N (**a**), P (**b**), and N/P ratio (**c**) accounting for climate (latitude, MAT, and MAP), plant functional types, and soil factors (N, P, and pH) globally. The full model (*R*^2^) represents the total variance explained, while individual predictors’ contributions to the model are also depicted. All metrics are normalized so that their sum equals 100%.

The hierarchical variation partitioning outcomes demonstrated that environmental and biological factors collectively explained 61%, 58%, and 41% of the variability in leaf litter N, P, and N/P ratio, respectively (Figure 6). The environmental and biological factors were classified into three variable groups, namely soil characteristics (pH and nutrients), climate-related gradients (latitude and climate conditions), and plant functional types. Among each variable group, climate-related gradients had the most substantial influence on leaf litter N/P ratio variations, while leaf litter P concentration variations were predominantly influenced by soil characteristics (encompassing soil nutrients and soil pH) (Figure 6). When considering each individual variable, plant functional type was the most important factor in explaining leaf litter N concentration (Figure 6a), followed by the climatic factors. Soil pH was the most parsimonious predictor in explaining leaf litter P concentration, followed by soil P and MAT (Figure 6b). For the leaf litter N/P ratio, the strongest predictor was latitude, followed by MAT and soil P (Figure 6c).

## 3. Discussion

Unlike previous regional-scale studies, our research is the first to our knowledge to conduct a quantitative review testing potential hypotheses regarding the patterns of leaf litter N, P, and N/P ratio.

### 3.1. Variation in Leaf Litter Stoichiometries Among Plant Functional Types

Significant differences in leaf litter stoichiometries occurred across different plant functional types in the global dataset. These differences were explained by the plant traits, including foliar longevity, morphology, or nutritional strategies [20]. For example, herbaceous plants were richest in both N and P, which generally resulted from their shorter life history, smaller size, and lower nutrient cycling than woody species [7,21]. Similarly, woody angiosperms, in general, had higher foliar element concentrations than gymnosperms, perhaps as a consequence of differences in their ability to absorb and sequester the nutrients into tissues [21,22].

### 3.2. Influence of Soil pH on Concentrations of Leaf Litter N and P

In the present study, both leaf litter N and P concentrations were positively correlated with soil pH. Positive relationships between soil pH and leaf litter N and P were also observed in other studies [9,13]. Soil pH is described as the “master soil variable” and has a great deal of influence on soil processes that affect plant growth and litter decomposition, e.g., the adsorption and solubility of mineral nutrients in soil [4,7]. As one of the controlling factors of nutrient availability, low soil pH values limit the availability of macronutrients in soil [4,13]. Moreover, plants struggle to absorb soil N and P under lower soil pH conditions [7,13].

### 3.3. Hypothetical Relationships Between Latitude and Leaf Litter N and P

The results of our study revealed that the *Species Composition hypothesis* best predicted the global pattern of leaf litter P, which aligns with results from studies on a more regional scale or of specific ecosystems [12,23]. The *Species Composition hypothesis* accurately predicted leaf litter P distribution based on plant functional type patterns. For instance, herbs with higher P content in leaf litter were less common in warm and humid zones (Figure S3, *p* < 0.01), contributing to the increase in leaf litter P at higher latitudes [21,23]. Authors of other studies also observed that broadleaf plants with lower P concentrations are mainly found in low-latitude areas compared to conifer trees [20]; thus, changes in species composition along latitudinal gradients influence leaf litter P patterns.

Unlike leaf litter P, all the potential hypotheses failed to predict the geographic patterns for leaf litter N. This also contrasts with previous findings regarding green leaf N, which was accurately predicted by both the *Temperature–Plant Physiology* and *Species Composition hypotheses* [9], possibly because of the disproportionate resorption of N during formation of leaf litter from fresh leaves [24]. Green leaf N tended to decline towards the equator (Figure S4), as predicted by both the *Temperature–Plant Physiology* and *Species Composition hypotheses*. Since leaf litter N concentration is always tightly aligned with green leaf N [9], its latitudinal pattern should follow that for green leaf N. However, due to the disproportionate resorption of N during the formation of leaf litter from fresh leaves, litter traits are not a mere legacy of fresh leaf traits [24]. N resorption efficiency (ratio of resorbed N to green leaf N during senescence) decreases drastically towards the equator, since high temperatures impaired the physiological mechanisms involved in nutrient resorption [20,24]. Eventually, the tropical plants exhibited N-poor green leaves but N-rich leaf litters, with N in leaf litters responding to latitude in a contrary manner to that of green leaves. Similar phenomena were also reported in other studies [9,24]. As a result, all the potential hypotheses which predicted elevated green leaf N towards polar zones failed to predict the geographic patterns for leaf litter N.

### 3.4. Factors Controlling Global Leaf Litter N and P

The results of our study revealed the key factors driving the global distribution of leaf litter stoichiometries. Among all influencing factors, plant functional type was identified as the most crucial determinant of leaf litter N levels, consistent with previous research [25]. Similar results were reported in a study of China’s forest ecosystems, where plant functional type was found to be the strongest predictor of leaf litter N [12]. In our study, variations in leaf litter N concentration across different plant functional types were mainly due to differences in leaf lifespan, morphology, and nutritional strategies [20]. For example, trees generally had lower N concentrations than herbaceous plants, likely because of their longer lifespan, larger size, and more efficient nutrient resorption [7,21]; woody angiosperms typically had higher leaf litter N levels than gymnosperms, possibly due to their greater nutrient uptake and storage efficiency [21]; and deciduous trees, with their shorter leaf lifespan, usually had higher leaf litter N concentrations than evergreen trees [4,7].

Soil pH was the primary factor influencing leaf litter P at the global scale in our study, and the predominance of soil pH in explaining the variation in leaf litter nutrients was also observed in other studies [9,12]. As discussed above, acidic soils were characterized by low availability of nutrients for plants. As one of the controlling factors of nutrient availability, low soil pH values limit the availability of macronutrients in soil [26,27,28]; therefore, the concentrations of P contained in litter usually increase with increasing soil pH.

Climatic factors appear to be dominant explanatory factors in the leaf litter N/P ratio [29], with our results on this factor being in line with those reported in a previous global meta-analysis [15,30]. Such a global pattern is related to the lower temperatures and smaller plant sizes in high-latitude ecosystems [31] and the very low N deposition and generally P-depleted soils in the tropics [7,15].

## 4. Materials and Methods

### 4.1. Data Collection

We systematically compiled a global leaf litter N and P database through extensive searches of the literature in Web of Science, Google Scholar, and China National Knowledge Infrastructure (CNKI) to identify publications from prior to 2024. Our search strategy employed Boolean operators combining (1) plant-related terms (“litter”) with (2) nutrient identifiers (“nitrogen” or “phosphorus”). The resulting database comprised 4657 observations of leaf litter N, P, and N/P spanning 1073 species across all vegetated continents, sourced from 170 peer-reviewed articles.

### 4.2. Criteria Used to Filter Collected Data

The database was assembled using rigorous selection protocols. First, we included only field-collected specimens from natural growing conditions [9]; second, for experimental studies involving environmental manipulations (e.g., temperature elevation, nutrient addition, or precipitation alteration), we exclusively extracted control group data to represent natural conditions [20,21]; third, we supplemented the nutrient data with corresponding site characteristics, including geographic coordinates, MAT (°C), and MAP (mm).

The compiled dataset exhibited broad biogeographical coverage, encompassing sites across 70 degrees of latitude with MATs ranging from subarctic (−5.4 °C) to tropical (33 °C) conditions and MAPs varying from arid (160 mm) to humid (4400 mm) regimes. While edaphic data availability was limited, we prioritized surface soil properties (typically 0–10 cm in depth) when reported [4]. Selected soil parameters (pH and nutrient concentrations) were included based on their established relationships with foliar elemental composition [17,18].

The dataset was stratified according to dominant vegetation classes. To evaluate the *Species Composition hypothesis* and to compare the leaf litter stoichiometries across different plant functional types, plant species were categorized into five functional groups based on established classification schemes: (1) non-seed plants (including moss and ferns), (2) grasses (monocots), (3) herbs (herbaceous dicots), (4) broadleaf woody (including shrubs and trees), and (5) conifers (gymnosperms) [9].

### 4.3. Statistical Analysis

Data normality and homogeneity of variance were verified using Kolmogorov–Smirnov and Levene’s tests, respectively (8). Due to skewed distributions in some variables, log_10_ transformation was implemented to improve model assumptions (Figure S5) [17]; we also implemented linear mixed-effects modeling to account for the hierarchical structure of our meta-analytic data [32]. The base model structure included response variables (leaf litter stoichiometry), covariates (latitude), fixed effects (climatic and edaphic factors), and random effects (site and plant functional type) [33]. Before comparison, heterogeneity tests were conducted for each variable. The differences between the leaf litter stoichiometries of the plant functional types were evaluated by LSD (least significant difference) at a 0.05 significance level, with site as the random effect, and we then conducted linear regression of stoichiometry against absolute latitude. To ascertain the relative importance of each potential explanatory variable in defining leaf nutrients, and to address collinearity among these variables, hierarchical variation partitioning was conducted for foliar N and P using the hier.part package [18]. All analyses were performed in R v3.4.3.

For N and P, we developed hypothesis-driven structural equation models (SEMs) using AMOS software (v18.0). The modeling framework incorporated latent variable specification, climatic factors (synthesizing latitude, MAT, and MAP), and plant functional types (aggregating five plant functional types). The model evaluation criteria were as follows: non-significant *χ*^2^ test (*p* > 0.05), *RMSEA* < 0.05, and *GFI*/*AGFI* > 0.90 [12,17]. This analytical approach allowed for the simultaneous examination of direct and indirect pathways among climatic, edaphic, and biological drivers of foliar chemistry.

## 5. Conclusions

In this study, we conduct an in-depth integrated analysis of climatic variables, soil properties, and plant functional types. The aim is to assess the validity of existing hypotheses and determine the key factors governing the global patterns of leaf litter N and P. Our results show significant differences in leaf litter N, P, and N/P ratio across different plant functional types in a global dataset. As proven by the SEMs, among the potential hypotheses, the *Species Composition hypothesis* is the most appropriate for leaf litter P, while all hypotheses fail to predict the geographic patterns for leaf litter N. Plant functional types, soil pH, and climatic factors are the strongest predictors of leaf litter N, P, and N/P ratio, respectively. Overall, the results of our study highlight the unique hypotheses and driving forces behind the global patterns of leaf litter stoichiometries.

These findings have implications for understanding ecosystem functions. Given that plant functional types, soil pH, and climatic factors are crucial for the global patterns of leaf litter stoichiometry, incorporating their changes into models can enhance our ability to predict biogeochemical cycles under climate change [34,35]; for instance, the invasion of woody plants into grasslands [36] may lead to a reduction in ecosystem-level leaf litter N. Moreover, we expect to test these potential hypotheses in individual climate zones and to include agricultural field conditions in further studies.

## Data Availability

Data are available upon request from the authors.

## Supplementary Materials

The following supporting information can be downloaded at: https://www.mdpi.com/article/10.3390/plants14213356/s1, Figure S1: Global variation in leaf litter stoichiometry; Figure S2: Latitudinal gradients in leaf litter stoichiometry of non-seed plants (a–c), grasses (d–f), herbs (g–i), broadleaf woody (j–l), and conifers (m–o) across global ecosystems; Figure S3: Distribution of plant functional types along mean annual temperature (MAT) and mean annual precipitation (MAP); Figure S4: Latitudinal gradients in green leaf N across global ecosystems; Figure S5: The distributions of latitude, climate, leaf litter stoichiometry, and soil characteristics; Table S1: Comparisons of leaf litter stoichiometry across different major plant functional types by linear mixed-effect models.
