# Supplementary material for: Potential Hypotheses Predicting Leaf Litter Nitrogen and Phosphorus Patterns at the Global Scale
Source: Plants (Basel). 2025 Nov 1;14(21):3356. doi: 10.3390/plants14213356 (PMC12608345; doi:10.3390/plants14213356)
Supplement: Supplementary file 1 [file plants-14-03356-s001.zip › plants-3753486-supplementary.pdf]

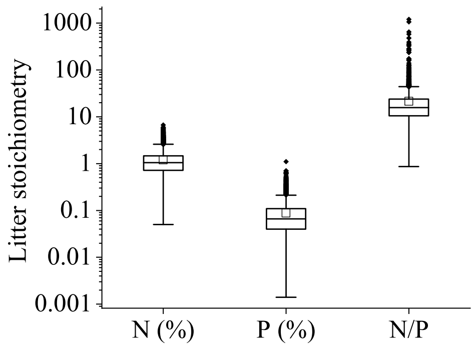

Figure S1. Global variation in leaf litter stoichiometry.

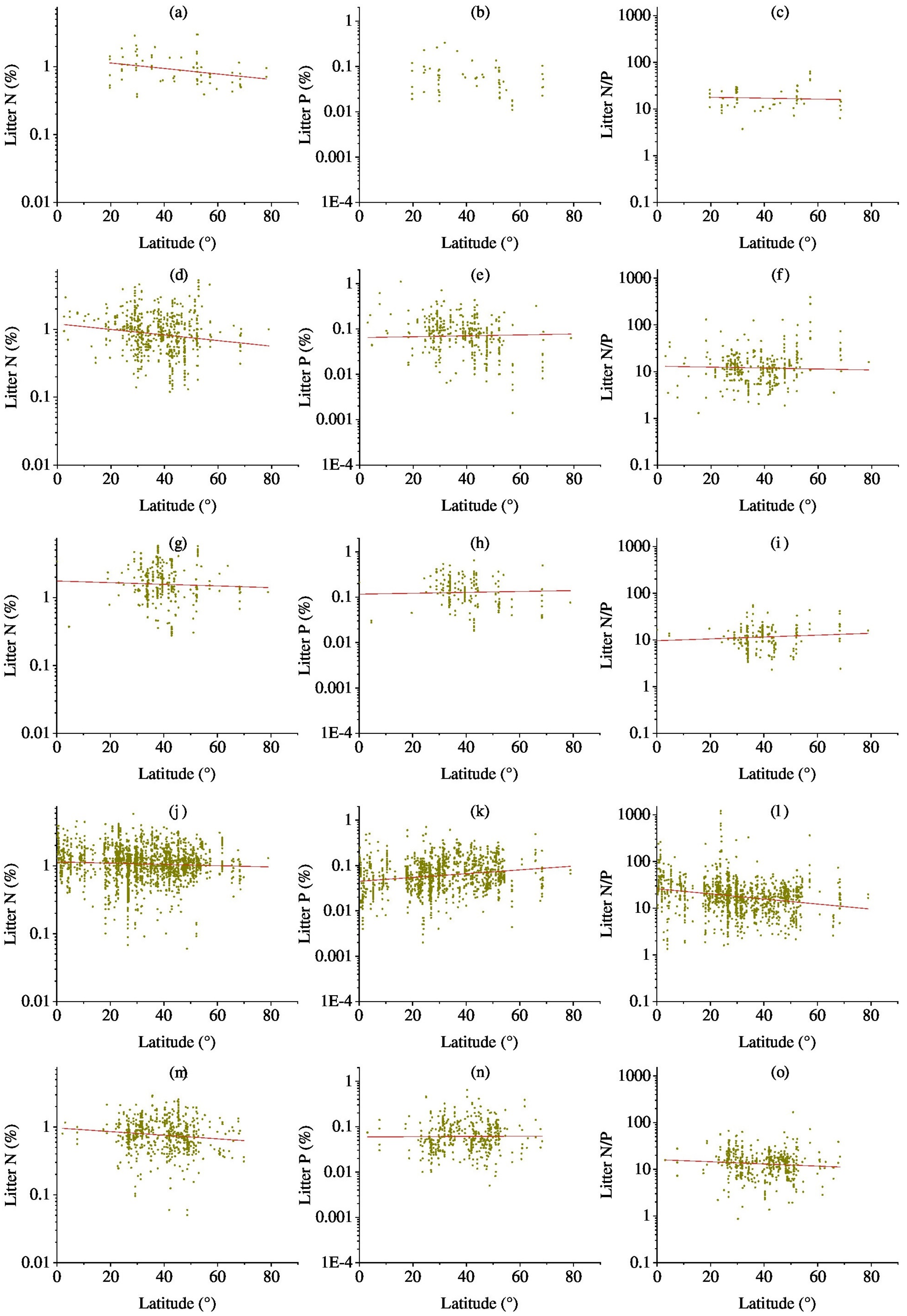

Figure S2. Latitudinal gradients in leaf litter stoichiometry of non-seed plants (a, b, and c), grasses (d, e, and f), herbs (g, h, and i), broadleaf woody (j, k, and l), and conifers (m, n, and o) across global ecosystems. All values were presented on log_{10}-transformed scales. Each data point represents site-level measurements of leaf litter chemistry plotted against absolute latitude.

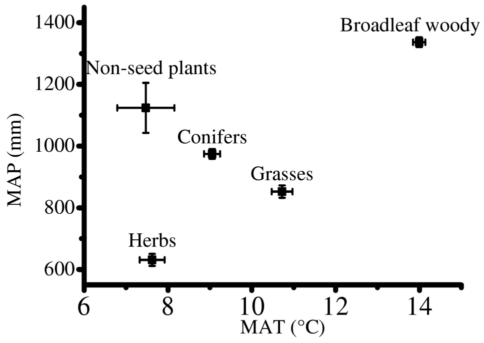

Figure S3. Distribution of plant functional types along mean annual temperature (MAT) and mean annual precipitation (MAP).

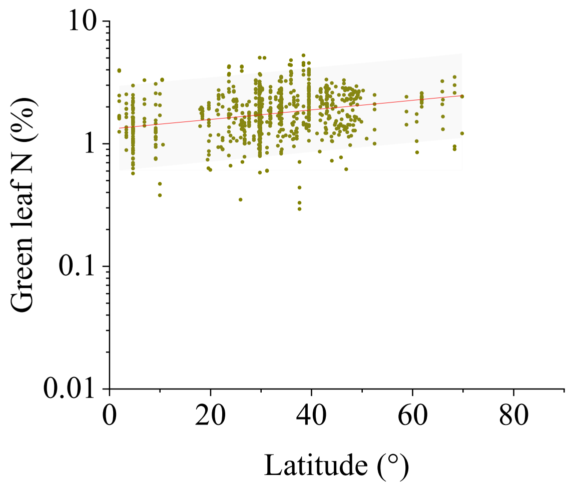

Figure S4. Latitudinal gradients in green leaf N across global ecosystems. All values were presented on log_{10}-transformed scales.

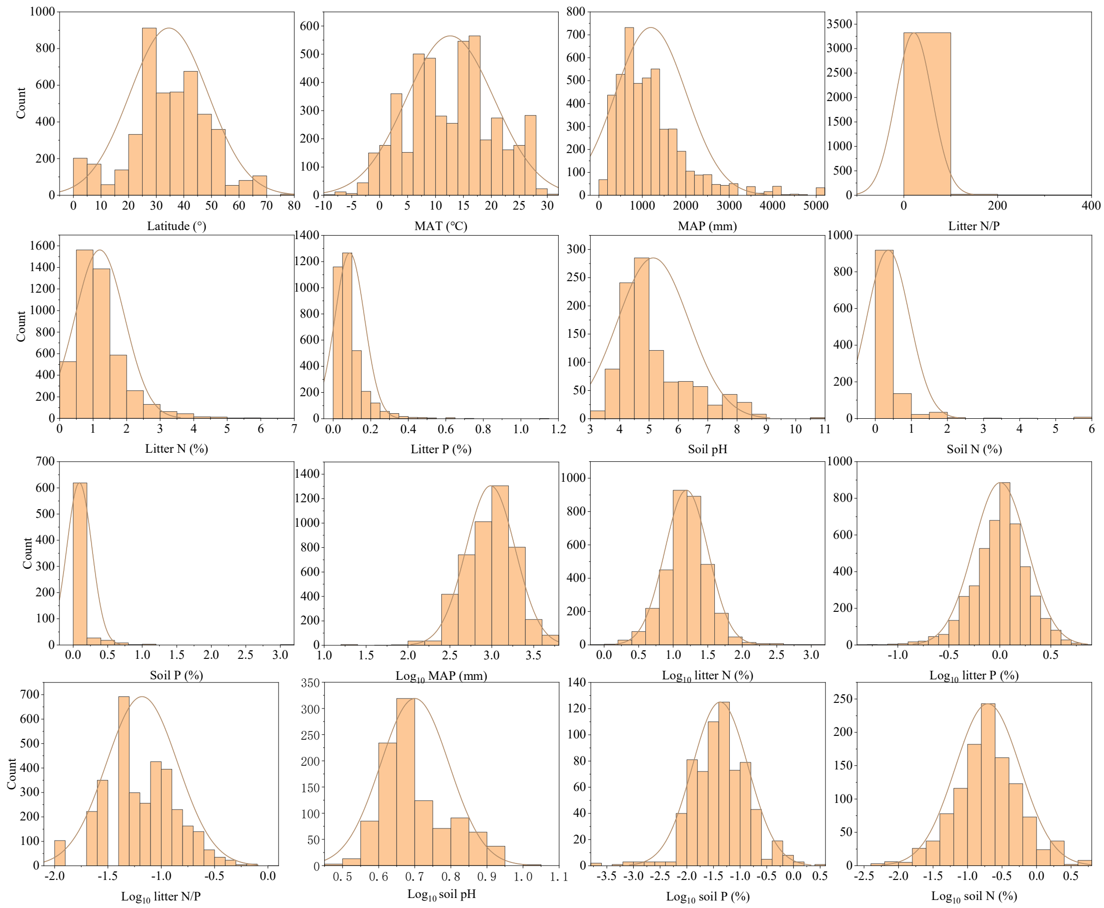

Figure S5. The distributions of latitude, climate, leaf litter stoichiometry, and soil characteristics. The curves indicate fitted normal curves.

Table S1. Comparisons of leaf litter stoichiometry across different major plant functional types by linear mixed-effect models.

**Leaf litter stoichiometry**	***F***	***p***
Leaf litter N	136	< 0.001
Leaf litter P	62	< 0.001
Leaf litter N/P ratio	12	< 0.001
